# Sequelae of Fetal Infection in a Non-human Primate Model of Listeriosis

**DOI:** 10.3389/fmicb.2019.02021

**Published:** 2019-09-11

**Authors:** Bryce Wolfe, Andrea R. Kerr, Andres Mejia, Heather A. Simmons, Charles J. Czuprynski, Thaddeus G. Golos

**Affiliations:** ^1^Department of Pathology and Laboratory Medicine, University of Wisconsin - Madison, Madison, WI, United States; ^2^Wisconsin National Primate Research Center, University of Wisconsin - Madison, Madison, WI, United States; ^3^Department of Comparative Biosciences, School of Veterinary Medicine, University of Wisconsin - Madison, Madison, WI, United States; ^4^Department of Pathobiological Sciences, School of Veterinary Medicine, University of Wisconsin -Madison, Madison, WI, United States; ^5^Department of Obstetrics and Gynecology, University of Wisconsin - Madison, Madison, WI, United States

**Keywords:** listeriosis, pregnancy, histopathology, cytokines, non-human primate, fetal infection

## Abstract

*Listeria monocytogenes* (Lm) is a common environmental bacterium that thrives on vegetation and soil matter, but can infect humans if contaminated food products are ingested, resulting in severe disease in immunosuppressed populations, including pregnant women and newborns. To better understand how the unique immunological milieu of pregnancy increases susceptibility to infection, we study listeriosis in cynomolgus macaques, a non-human primate that closely resembles humans in placentation and in the physiology, and immunology of pregnancy. Non-human primates are naturally susceptible to Lm infection, and spontaneous abortions due to listeriosis are known to occur in outdoor macaque colonies, making them ideal models to understand the disease pathogenesis and host-pathogen relationship of listeriosis. We have previously shown that Lm infection in the first trimester has a high rate of miscarriage. This study expands on our previous findings by assessing how the quantity of Lm as well as stage of pregnancy at the time of exposure may influence disease susceptibility. In the current study we inoculated a cohort of macaques with a lower dose of Lm than our previous study and although this did not result in fetal demise, there was evidence of *in utero* inflammation and fetal distress. Animals that were reinfected with an equivalent or higher dose of the same strain of Lm resulted in approximately half of cases continuing to term and half ending in fetal demise. These cases had inconsistent bacterial colonization of the fetal compartment, suggesting that Lm does not need to directly infect the placenta to cause adverse pregnancy outcomes. Timed surgical collection of tissues following inoculation demonstrated that transmission from mother to fetus can occur as soon as 5 days post-inoculation. Lastly, third trimester inoculation resulted in pregnancy loss in 3 out of 4 macaques, accompanied by characteristic pathology and Lm colonization. Collectively, our studies demonstrate that common laboratory culture tests may not always recover Lm despite known maternal ingestion. Notably, we also find it is possible for maternal infection to resolve in some cases with no discernible adverse outcome; however, such cases had evidence of a sterile intrauterine inflammatory response, with unknown consequences for fetal development.

## Introduction

Listeriosis remains a public health concern for pregnant individuals, despite efforts in surveillance, reporting, public awareness campaigns, and implementation of safety measures by industry and agriculture (Tappero et al., 1995; Pohl et al., 2019). A foodborne disease caused by the bacterium *Listeria monocytogenes* (Lm), listeriosis can result in miscarriage, stillbirth, preterm birth, or neonatal infection (Craig et al., 2019). Lm is able to survive in a wide range of environments, including at refrigeration temperature. Common sources of infection include unpasteurized dairy products and ready-to-eat foods, although less common items such as caramel-coated apples have been responsible for human outbreaks (Glass et al., 2015). It can lead to a particularly insidious disease because symptoms may be mild, mistaken for the flu, or masked by morning sickness, resulting in delayed diagnosis and treatment. The United States' Centers for Disease Control and Prevention (CDC) reports that 1 in 6 cases of listeriosis in the U.S. is associated with pregnancy (Centers for Disease Control and Prevention, 2019) and that nearly one quarter of pregnancy-associated cases result in fetal loss or death of the newborn (Centers for Disease Control and Prevention, 2016). Most data concerning the course and nature of human infection come from retrospective clinical cases, identified based on symptoms, positive bacterial cultures, or adverse pregnancy outcomes. The first prospective clinical study, the Multicentric Observational NAtional Study on LISteriosis and ListeriA (MONALISA), found that the disease burden of listeriosis is higher than previously estimated: particularly, that more than 80% of infected mothers experienced major fetal or neonatal complications, and that the rate of fetal loss was significantly greater at <29 weeks of gestation (Charlier et al., 2017). Underreporting of pregnancy-associated listeriosis is very likely due to undiagnosed asymptomatic illness, unreported/unrecognized early miscarriage, misdiagnosed stillbirths, and difficulty in positively identifying Lm by standard laboratory culture, and Gram stain (Kylat et al., 2016).

Our current study addresses these limitations using a macaque model of infection, which allows for experimental manipulation, including a predetermined dose and timing of exposure, in a system highly relevant to the unique structure and physiology of human pregnancy (Lamond and Freitag, 2018; Lowe et al., 2018). Previously we found that dams given an inoculum of 10^7^ CFUs Lm in the first trimester of pregnancy resulted in rapid infection and fetal demise, accompanied by diffuse bacterial colonization of the maternal-fetal interface (Wolfe et al., 2017). The objectives of the current study were to determine how a milder exposure changes the disease trajectory and outcome, and if increasing gestational age or previous infection influences susceptibility. We gave dams a single dose of 10^6^ CFUs Lm in the first trimester. Exposure to <10^7^ CFUs Lm in a single dose did not result in acute pregnancy loss. In subsequent pregnancies, we gave dams an equivalent or higher dose of Lm than previously administered. We find that re-exposure following a previous infection resulted in a lower rate of bacterial colonization, but could still induce adverse pregnancy outcomes associated with a sterile inflammatory response in the placenta and fetus. Because the maternal-fetal interface is not in a static state as pregnancy progresses, we next assessed third trimester infection to determine if increasing gestational age may enhance or mitigate Lm virulence. Our results demonstrate that infection with 10^7^ CFUs Lm in late gestation results in a lower bacterial burden and slightly higher rate of fetal survival compared to the same dose in early gestation (33 vs. 20%, respectively), but does not mitigate against adverse pregnancy outcomes, which remain severe and can occur in as few as 4 days following exposure.

## Materials and Methods

### Ethics Statement

The cynomolgus macaques (*Macaca fascicularis*) in this study were cared for by the staff at the Wisconsin National Primate Research Center (WNPRC) according to the regulations and guidelines of the University of Wisconsin Institutional Animal Care and Use Committee which approved this study, and adheres to principles described in the National Research Council's Guide for the Care and Use of Laboratory Animals.

### Animals and Breeding

Adult female cynomolgus monkeys maintained at the WNPRC were cohoused with compatible males and observed daily for menses and copulation. Pregnancy was detected by ultrasound examination of the uterus approximately 18 to 20 days following the predicted day of ovulation. The day of gestation when pregnancy was detected was estimated based on previous experience and published data describing cynomolgus macaque fetal size during gestation (Tarantal and Hendrickx, 1988). Ultrasound examination of the uterus was done weekly or biweekly to monitor placental and fetal growth until the day of Lm inoculation, and every 2–3 days or daily after inoculation to assess fetal vital signs.

### Inoculation With *L. monocytogenes*

At varied days of gestation in the first trimester (between days 36 to 50) or third trimester (between days 110 to 135 [full term is day 165]), monkeys were sedated, the uterus was examined by ultrasound to confirm a viable pregnancy, and a single dose between 10^6^ and 10^8^ colony forming units (CFUs) log-phase cells of strain LM2203 (serotype 4b, derived from an outbreak of listeriosis among pregnant women in Winston-Salem, North Carolina in the year 2000 MacDonald et al., 2005), were administered in 10 ml of whipping cream via a soft intragastric feeding tube as previously described (Smith et al., 2003). For each inoculation, Lm was cultured at 37°C in Tryptic Soy Broth (Becton Dickinson, Sparks, MD). 500 ul of the inoculum was diluted in phosphate-buffered saline (PBS; Catalog #P5368, Sigma-Aldrich, St. Louis, MO), plated on Trypticase soy agar with 5% sheep blood (Becton Dickinson, Sparks, MD), and incubated at 37°C to confirm the dose given to each animal as CFUs of Lm per milliliter. Six monkeys were given a whipping cream inoculum without Lm in an identical manner to be included as uninfected controls.

### Fecal Shedding

**Figure 1:**
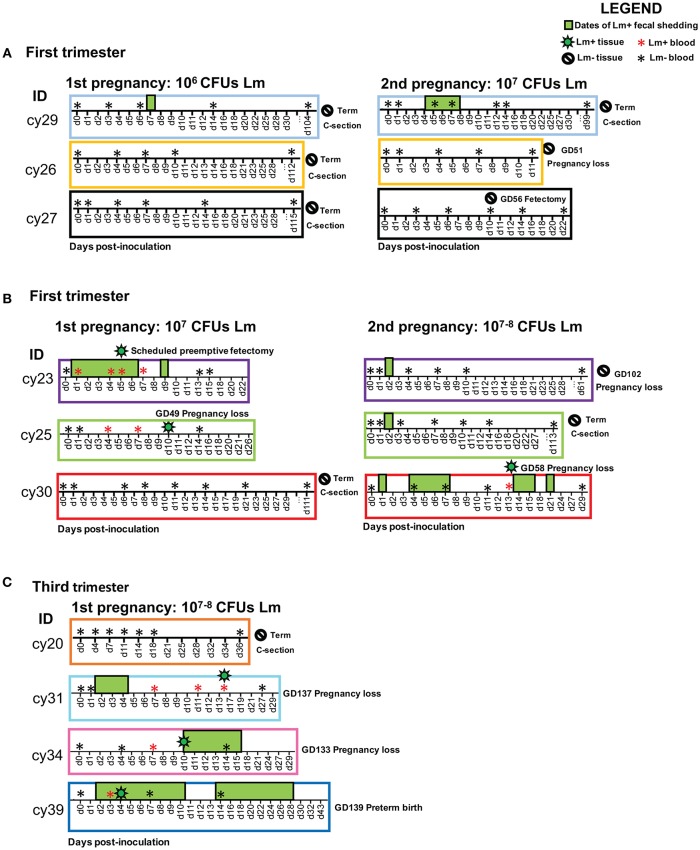
Experimental timelines for each cohort showing animal ID, sample collections, and pregnancy outcome. The x-axis displays post-inoculation days. The following symbols indicate events on the timeline: positive blood culture (red asterisk), negative blood culture (black asterisk), positive tissue culture (green bacteria), negative tissue culture (negative sign), and positive fecal culture (green box). Fecal samples were collected on each date shown on the timeline; dates without a green box denote negative fecal cultures, and absent dates indicate that no samples were collected. Overlapping symbols indicate concurrent events. **(A)** First trimester cohort: 10^6^ CFUs Lm initial dose; 10^7^ CFUs Lm reexposure. **(B)** First trimester cohort: 10^7^ CFUs Lm initial dose; 10^7^ CFUs Lm reexposure. **(C)** Third trimester cohort: 10^7^–10^8^ CFUs Lm dose.

Before and after inoculation, fecal samples were collected from cage pans to monitor fecal shedding of Lm. Schedules of sample collection are described in the legend to Figure 1. Serial dilutions in PBS of fecal samples were plated in duplicate on modified Oxford agar plates (Kang and Fun, 1999) and the number of colonies was determined using ImageJ colony-counting software (https://imagej.nih.gov/ij/plugins/colony-counter.html) after 24 to 48 h of incubation at 37°C.

### Bacteremia Monitoring

Peripheral blood samples were collected periodically for aerobic and anaerobic cultures to detect bacteremia as previously described (Lancaster, 2015) and processed on a BD Bactec 9,050 blood culture system (Becton Dickinson Diagnostic Systems, Sparks, MD) in the Clinical Pathology Laboratory at the University of Wisconsin—Madison School of Veterinary Medicine. Bactec Peds Plus/F blood culture bottles aseptically inoculated with 3 ml sample per bottle were incubated at 35°C until a positive signal was observed or for a maximum of 5 days. Bottles that did not signal positive at the end of 5 days were Gram-stained and sub-cultured to two chocolate agar plates (Remel, Lenexa, KS). Subcultures were incubated for 24–48 h aerobically at 35°C in 5% CO2 and anaerobically in an anaerobic chamber (SHEL LAB, Cornelius, OR) at 35°C in 80% N2, 5% H2, and 5% CO2 for verification of true or false negativity. Positive blood cultures were Gram-stained and subcultured to a blood agar plate supplemented with 5% sheep blood, chocolate agar, eosin methylene blue agar, and Columbia nalidixic acid agar (Remel, Lenexa, KS), and incubated at 35°C for 24–28 h in 5% CO2. An anaerobic subculture was also performed and incubated in an anaerobic chamber for 24–48 h as indicated above. Recovered isolates were identified by matrix-assisted laser desorption-ionization time-of-flight (MALDI-TOF) mass spectrometry (Bruker Daltonics, Billerica, MA). Sample extraction and strain identification was performed following the manufacturer's instruction. A score of >2 indicated secure genus and probable species identification.

### Surgery and Tissue Processing

Ultrasound examination of the uterus was done 1 to 3 times per week after Lm dosing to monitor fetal well-being and confirm fetal heartbeat and umbilical blood flow. When fetal demise was indicated by absence of heartbeat, fetal and maternal tissues were promptly collected at laparotomy. The entire conceptus (decidua, placenta, fetal membranes, umbilical cord, amniotic fluid, and fetus) was removed by uterotomy. These were survival surgeries for the dams. The fetus was dissected into ~4-mm coronal segments, and alternating segments were fixed and embedded for histology (see below), or homogenized for bacteriological analysis on blood agar plates as previously described (Poulsen et al., 2013).

### Histology and Histopathological Scoring

Dissected tissues were fixed in 2 to 4% paraformaldehyde for 24 to 72 h, rinsed in PBS, and stored in 70% ethanol until processed and embedded in paraffin. Paraffin sections were stained with hematoxylin and eosin (H&E) and Gram-stained using standard methods. Lm was confirmed by chromogenic immunohistochemistry with *Listeria* O antiserum (Difco Laboratories' rabbit polyclonal antibody against the somatic O antigen of *Listeria* serogroup 4). Stained sections were then examined and scored by WNPRC veterinary pathologists. Gram-stained slides were digitally scanned by the Wisconsin State Lab of Hygiene and analyzed using Aperio eSlide Manager with ImageScope 12.4 software (Leica Biosystems Inc. Buffalo Grove, IL). Scatterplot graphs of histological scores were prepared using GraphPad Prism version 8.0 (GraphPad Software, La Jolla, CA).

### Cytokine/Chemokine Expression

Homogenized tissue samples were analyzed for cytokine expression by Luminex multiplex assay (Luminex Corporation, Austin, TX) with an NHP-validated kit containing CCL5/Regulated on Activation, Normal T cell Expressed and Secreted (RANTES), interleukin-1 beta (IL1b), IL-1 receptor antagonist (IL1ra), IL-2, IL-4, IL-6, IL-8, IL-10, IL-12, IL-13, IL-15, IL-16, IL-17 alpha, IL-18, IL-21, IL-23, IL-33, fibroblast growth factor 2 (FGF-2), granulocyte colony-stimulating factor (G-CSF), granulocyte macrophage-colony stimulating factor (GM-CSF), granzyme A, interferon gamma (IFN-g), monocyte chemoattractant protein (MCP)-1, monocyte inflammatory protein (MIP)-1alpha, MIP-1beta, perforin, soluble CD40 Ligand (sCD40L), soluble FAS ligand (sFASL), tumor necrosis factor alpha (TNFa), and vascular endothelial growth factor (VEGF) (Milliplex Non-Human Primate Cytokine and Chemokine Panel, Millipore, Billerica, MA). Cytokine and chemokine concentrations were quantified using the Bioplex 200 system (Biorad, Hercules, CA). Concentrations were normalized to the protein levels in each homogenate using a Thermo Scientific Pierce Micro BCA Protein Assay Kit (Catolog #23235, Thermo Fisher Scientific, Waltham, MA). Values were log-transformed and hierarchical clustering of principle components was performed using ClustVis (Metsalu and Vilo, 2015).

## Results

### Pregnancy Outcomes With 10^6^ Lm

**Figure 2:**
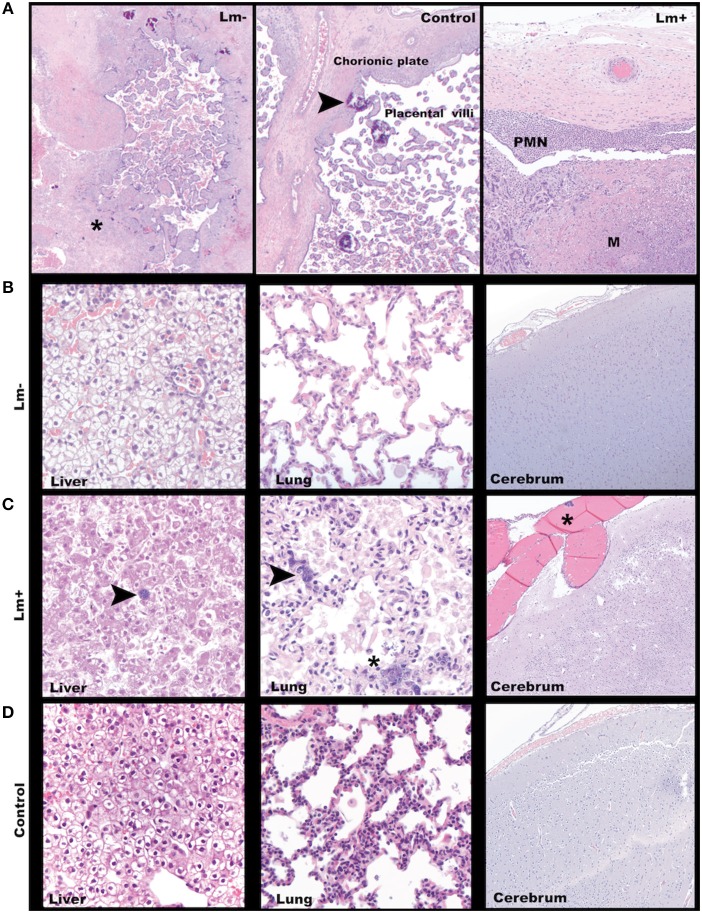
Third trimester histology. **(A)** Maternal-fetal interface. Left: A focus of avascular villi entrapped in fibrin (asterisk) in a placenta from a dam exposed to Lm but without discernible infection. Cy29.1 H&E, 2x. Center: Normal placenta with incidental multifocal mineralization (arrowhead). Cy33. H&E, 40x. Right: Lm-infected placenta with acute subchorionic neutrophilic (PMN) inflammation and intervillous microabscesses (M). Cy31. H&E, 2x. **(B)** Representative tissue findings in Lm-inoculated animals who did not show clinical signs of infection. Left: Fetal liver with mild diffuse subcapsular neutrophil infiltration, intravascular and perivascular neutrophils. Cy27.1. H&E 40x. Center: Fetal lung with rare squamous cells in alveoli. Cy26.1. H&E 40x. Right: Fetal cerebrum with no significant findings Cy26.1. H&E, 10x. **(C)** Representative tissue findings in Lm-infected animals. Left: Lm+ Fetal liver with intralesional bacteria (arrow). Cy31. H&E, 10x. Center: Lm+ fetal lung with intravascular bacteria (arrow) and alveolus with luminal bacteria, neutrophils, squamous cells, and cellular debris. Cy31. H&E, 40x. Right: Lm+ fetal cerebrum with an intravascular bacterial embolus (asterisk). Cy31. H&E, 10x. **(D)** Representative tissue findings in control animals. Left: Control fetal liver. Cy33. H&E, 40x. Center: Control fetal lung. Occasional interalveolar squamous cells are an incidental finding commonly seen in cesarean deliveries. Cy33. H&E, 40x. Right: Control fetal cerebrum. Cy33. H&E, 10x.

**Figure 3:**
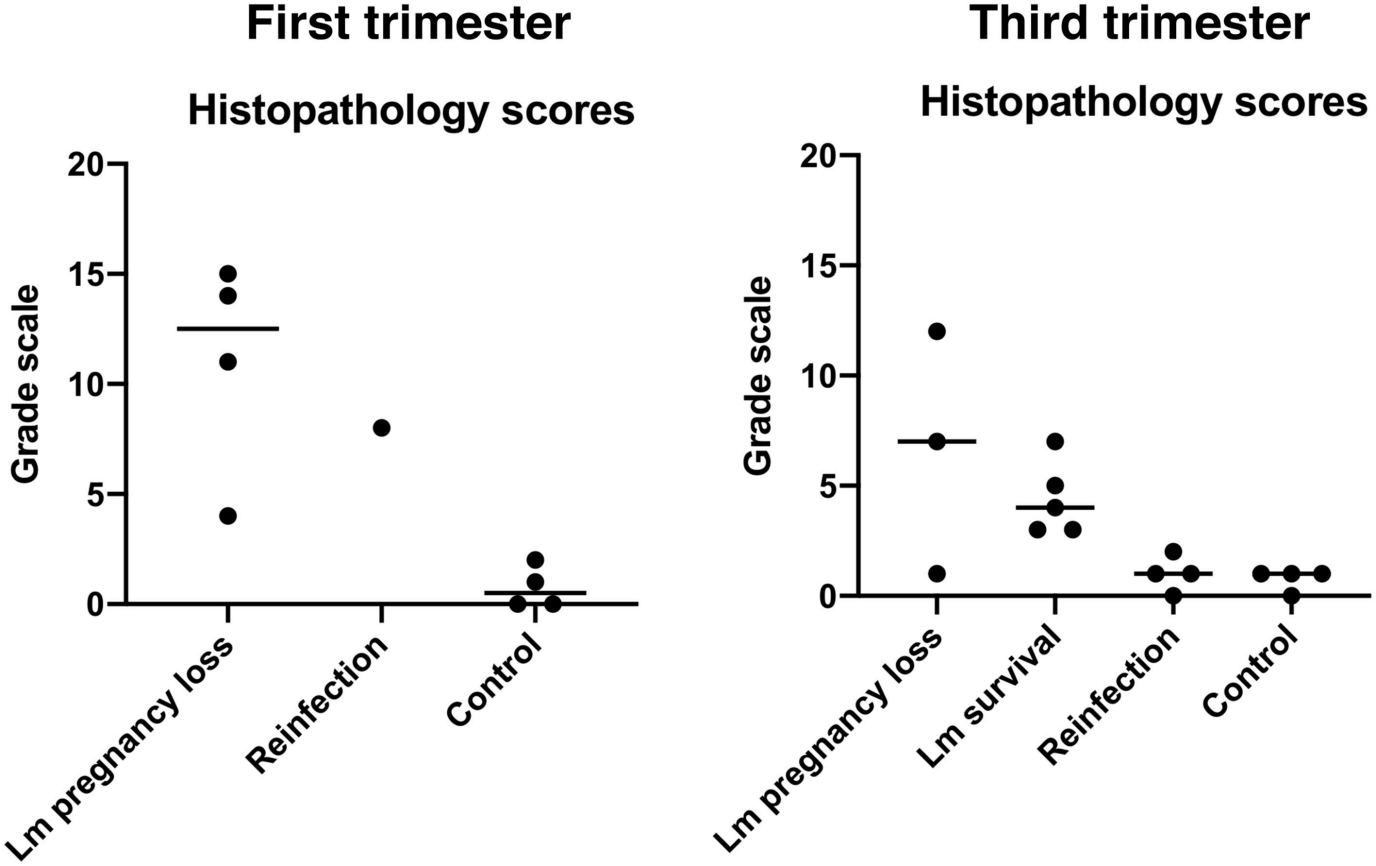
Histopathology scores. Each dot represents the final score from a single case. Final scores were calculated from the sum of abnormal histological findings noted in both fetal and maternal-fetal interface tissues. Central black lines indicate the average score of a group.

**Figure 4:**
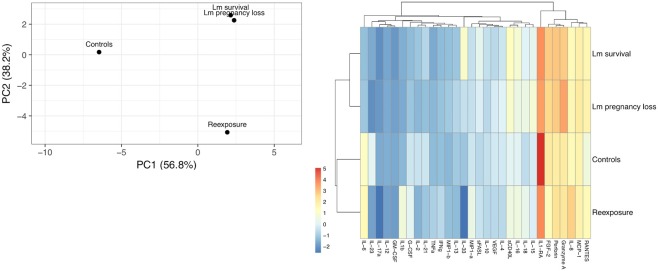
Cytokine cluster analysis of third trimester placenta samples. **(A)** Principle component analysis based on the variation between subgroups. Original values were ln(x + 1)-transformed. Singular value decomposition with imputation was used to calculate principal components. X and Y axes show principal component 1 and principal component 2 that explain 56.4 and 37.2% of the total variance, respectively. **(B)** Heat map showing relative expression of all 30 cytokines assayed. Original values were ln(x + 1)-transformed. Pareto scaling was applied to rows. Both rows and columns were clustered using Manhattan distance and average linkage. Dendrograms indicate tightness of clusters.

All three dams continued to term with no evident fetal infection and no evident colonization of the maternal reproductive tract (Table 1; Figure 1A). There was sporadic maternal fecal shedding, but none of the dams showed signs of illness such as inappetence, fever, or bacteremia. Tissues collected at cesarean section appeared grossly normal in all cases. Microscopically, however, all cases had histologic signs of inflammation, including decidual fibrinoid necrosis, neutrophilic villitis with villous necrosis, neutrophilic chorioamnionitis, fetal hepatitis, and increased numbers of intra-alveolar squamous cells and cellular debris consistent with fetal distress *in utero* (Figures 2A,B). The placenta from case cy29.1 also had evidence that basal plate infarction and bleeding into the intervillous space had occurred earlier in gestation, with focal but extensive areas of organized fibrin and hemosiderin deposition. Cases cy29.1 and cy26.1 had moderate numbers of lymphocytes and plasma cells within the basal plate and placental villi, which is indicative of a chronic inflammatory response. After grading for extent and severity of histopathology, all cases from this cohort scored higher than age-matched controls (Figure 3: Lower dose vs. Control). Consistent with this trend, placenta samples from this cohort had increased levels of several pro-inflammatory cytokines, including RANTES, perforin, and granzyme A. Anti-inflammatory IL-1RA, which antagonizes IL1b, was highest in controls and lowest with Lm infection. Placentas from pregnancies that received Lm but continued to term C-section had levels of IL-1RA higher than samples from Lm+ cases of pregnancy loss, but lower than age-matched controls (Figure 4).

**Table 1:** Overview of treatment groups and outcomes.

**Treatment**	**Trimester**	**Cohort**	**Outcome**	**ID**	**Gestation at inoculation**	**Maternal age**	**Maternal weight (kg)**	**Confirmed total dose Lm**	**Dose by weight (Lm/kg)**	**Peak maternal temperature (^**°**^F)**	**Gestation at collection**	**Duration**	**Fetal sex**	**Fetal weight** **(kg)**
Lm	1	10^6^, first exposure	Fetal survival	cy29.1	44	6	3.74	1.64E+06	4.39E+05	101	148	104	M	0.32
Lm	1	10^6^, first exposure	Fetal survival	cy26.1	40	7	4.2	2.80E+06	6.67E+05	100.5	152	112	F	0.34
Lm	1	10^6^, first exposure	Fetal survival	cy27.1	39	7	4.5	2.60E+06	5.78E+05	101.4	154	115	F	0.45
Lm	1	10^7^, first exposure	Pregnancy loss	cy19.1	40	9	5.74	2.47E+07	4.30E+06	102.1^*^	48	8	x	0.004
Lm	1	10^7^, first exposure	Pregnancy loss	cy21.1	36	6	3.35	1.23E+07	3.67E+06	101.4	50	14	x	0.019
Lm	1	10^7^, first exposure	Pregnancy loss	cy23.1	41	7	5.03	1.52E+07	3.02E+06	101	46	5	x	(–)
Lm	1	10^7^, first exposure	Pregnancy loss	cy25.1	39	6	3.32	1.60E+07	4.82E+06	102.9^*^	50	11	x	(–)
Lm	1	10^7^, first exposure	Fetal survival	cy30.1	43	13	3.45	1.65E+07	4.78E+06	101.1	154	111	M	0.33
Lm	1	10^7^, re-exposure	Fetal survival	cy29.2	47	7	4.66	1.34E+07	2.88E+06	101	145	98	F	0.33
Lm	1	10^7^, re-exposure	Pregnancy loss	cy26.2	40	8	4.78	2.00E+07	4.18E+06	98.9	51	11	x	(–)
Lm	1	10^7^, re-exposure	Timed fetectomy	cy27.2	46	8	4.44	1.13E+07	2.55E+06	98.6	56	10	x	0.0101
Lm	1	10^7^, re-exposure	Pregnancy loss	cy23.2	41	8	5.2	1.20E+07	2.31E+06	101.2	102	61	x	0.098
Lm	1	10^7^, re-exposure	Fetal survival	cy25.2	39	7	3.9	2.00E+07	5.13E+06	99.7	153	114	M	0.34
Lm	1	10^8^, re-exposure	Pregnancy loss	cy30.2	45	14	4.16	1.40E+08	3.37E+07	99.1	58	13	x	0.0115
Lm	3	10^7^, first exposure	Fetal survival	cy20.1	110	5	5.92	1.60E+07	2.70E+06	100.3	146	36	M	0.34
Lm	3	10^7^, first exposure	Pregnancy loss	cy31.1	123	6	4	1.60E+07	4.00E+06	101	137	14	M	0.27
Lm	3	10^7^, first exposure	Pregnancy loss	cy34.1	123	9	5.34	1.12E+07	2.10E+06	96.6	133	10	M	0.21
Lm	3	10^8^, first exposure	Pregnancy loss	cy39.1	135	5	3.86	2.33E+08	6.04E+07	101.2	139	4	M	0.25
None	1	Control	Normal	cy24c	42	8	4.38	n/a	n/a	99.3	57	15	x	0.008
None	1	Control	Normal	cy27c	41	6	4.14	n/a	n/a	99.9	51	10	x	0.006
None	1	Control	Normal	cy35c	46	5	2.68	n/a	n/a	99.5	51	5	x	0.003
None	1	Control	Normal	cy26c	38	6	3.86	n/a	n/a	99.7	55	17	x	0.02
None	3	Control	Normal	cy35c	141	6	3.54	n/a	n/a	98.6	154	13	M	0.31
None	3	Control	Normal	cy33	124	10	8.15	n/a	n/a	97.9	152	28	F	0.34
None	3	Control	Normal	cy38	115	5	4.03	n/a	n/a	99.9	137	22	F	0.31
None	3	Control	Normal	cy31c	133	9	4.36	n/a	n/a	98.1	137	4	M	0.296

*Cynomolgus macaque females reach maturity around age 4, are fertile until approximately age 24, and have an average lifespan of 30 years*.

* *Maternal fever*.

*x, too early in gestation to determine*.

*(–), not recorded*.

### Pregnancy Outcomes Following Re-infection

**Figure 5:**
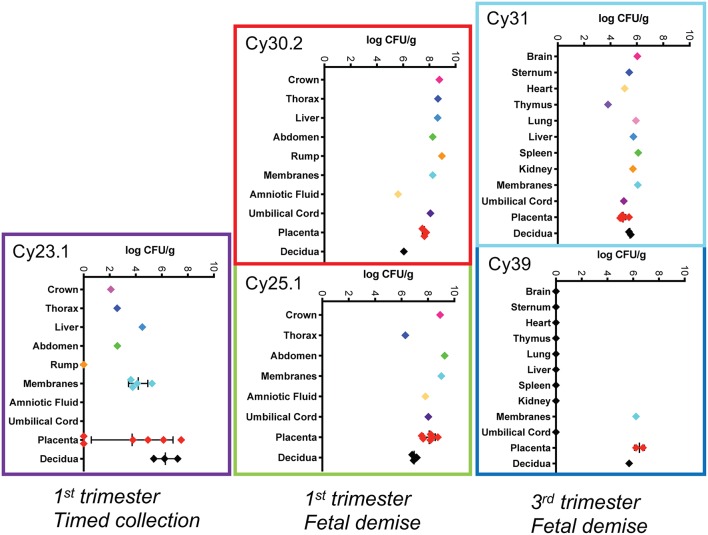
Tissue bacterial burden Bacterial burden of Lm from individual pregnancies. The y-axis shows tissues and the x-axis shows the bacterial burden in colony forming units per gram (CFUs/g) in log scale for each tissue. Central black lines indicate the mean CFUs/g for replicate samples.

**Figure 6:**
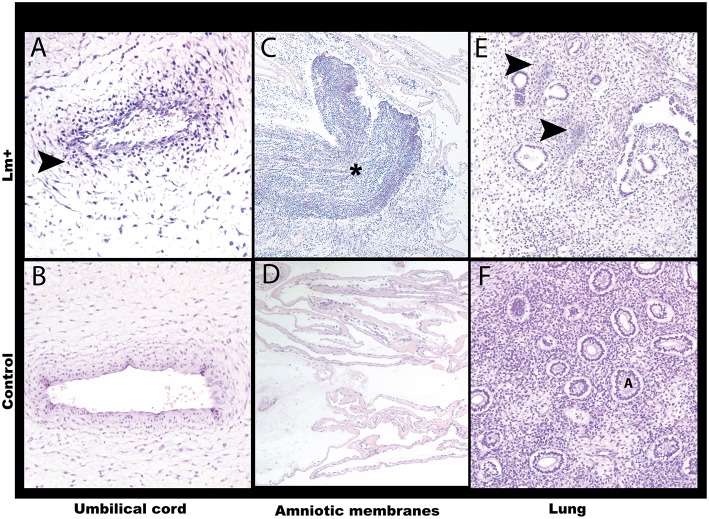
Histology of the first trimester Lm infection. **(A)** Umbilical cord. Neutrophilic umbilical arteritis. Arrow points to clustered neutrophils. Cy30.2. H&E, 20x. **(B)** Control umbilical cord. Cy26c H&E, 20x. **(C)** Amniotic membranes with Gram-positive bacteria and neutrophilic infiltrate (asterisk). Cy30.2. H&E, 4x. **(D)** Control amniotic membranes. Cy26c H&E, 20x. **(E)** Fetal lung with listerial infiltration of pulmonary interstitium between alveoli. Arrows point to bacterial foci. Cy30.2 H&E, 20x. **(F)** Control fetal lung. A indicates alveolus. Cy26c H&E, 20x.

Inoculation with the same strain of Lm in a subsequent pregnancy resulted in fetal demise in 3 of 5 dams: cy26.2 at gestation day (gd)51 (11 days post-inoculation), cy30.2 at gd58 (13 days post-inoculation), and cy23.2 at gd102 (61 days post-inoculation) (Figure 1B). In this cohort, two received an Lm dose equivalent to their first exposure (cy26.2, cy29.2), and three received a higher dose (cy23.2, cy25.2, cy30.2) (Table 1). We were unable to culture bacteria (Lm) from the maternal-fetal interface in two cases (cy26.2 and cy23.2), and one of the dams (cy26.2) had no detectable Lm shedding or signs of infection prior to first trimester miscarriage. In this case (cy26.2), placental and fetal tissues were not available for analysis due to maternal placentophagy following spontaneous abortion. Cy23.2 had Lm-positive maternal fecal shedding and miscarried in the second trimester. Tissues examined from cy23.2 demonstrated mild histopathology, with multifocal neutrophilic villitis in the placenta and diffuse lymphoplasmacytic deciduitis. The fifth dam (cy30.2) received a higher dose in her second pregnancy (10^8^ CFUs) and spontaneously aborted at gd58 (13 days post-inoculation), accompanied by fecal shedding, maternal bacteremia, and tissue colonization and histopathology characteristic of listeriosis (Figures 1B, 5, 6). The remaining two cases, cy29.2 and cy25.2, continued uneventfully to term. On histological examination, the placenta from cy29.2 showed basal plate infarcts with hemosiderin, indicating prior hemorrhage, and the placenta from cy25.2 had focal villous infarcts with lymphoplasmacytic deciduitis. Interestingly, placenta samples from this cohort did not cluster with control samples by PCA plot and showed a shift in cytokine expression distinct from the other cohorts (Figure 4).

### Timed Surgical Collection Following Inoculation to Assess Timing of Colonization

**Figure 7:**
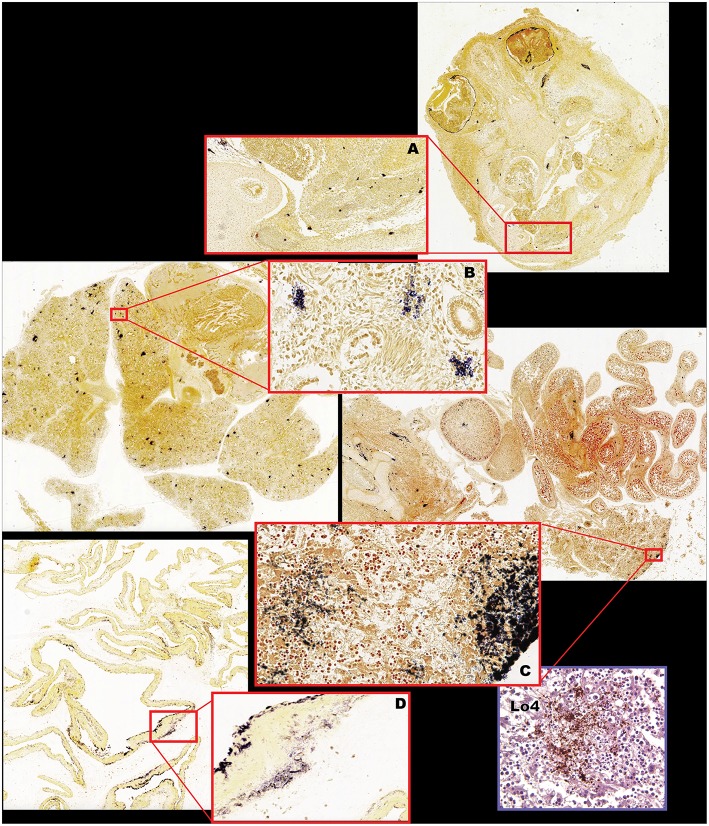
Fetal bacterial distribution with Lm infection in the first trimester (Cy25.1). **(A)** Fetal cranium with large clusters of Gram-positive rods within the ocular musculature, diffusely within the nasal sinus and nasal cartilage, multifocally within the neural tissue, periosteum of the skull, and within the dermis, and subcutaneous tissues. Gram stain, 2x (inset 10x). **(B)** Fetal lung with multiple foci of intravascular bacteria. Gram stain, 2x (inset 40x). **(C)** Fetal liver, vertebrae, intestines, kidney, and adrenal gland, with multiple foci of bacterial colonization of the kidney, adrenal gland, intestinal serosa, hepatic capsule and interstitium, periosteum, and perichondrium. Gram stain, 2x (inset liver 40x). **(D)** Fetal membranes (chorioamniotic membranes) with multiple foci of Gram-positive bacteria. Gram stain 2x (inset 10x). Inset (Lo4): Immunohistochemical staining of fetal liver confirming Lm-positive foci.

We performed pre-emptive surgery and sample collection shortly following inoculation to assess early events at the maternal-fetal interface. We assessed first time exposure (cy23.1) and re-exposure (cy27.2). Tissues collected at post-inoculation day 5 (cy23.1) showed that replicate decidua biopsies had a consistently high bacterial burden (5–7 log CFUs/g) whereas replicate placenta biopsies had more variable colonization (0–8 log CFUs/g). Of these, 2 of 6 replicates yielded no culturable Lm. There was moderate colonization of the amniotic membranes (3–6 log CFUs/g), and no culturable bacteria in the amniotic fluid (Figures 1B, 5), suggesting that surgery had interrupted colonization of the fetal compartment. Once the placenta has been breached, Lm characteristically colonizes highly vascular tissues of the fetus including developing bone, lung, spleen, and liver (Figure 7). The most heavily infected fetal organ in case cy23.1 was liver, with a bacterial burden equivalent to that of the amniotic membranes (Figure 5). All fetal tissues in this case had a bacterial burden several logs lower than cases which had fetal death *in utero* and an incubation duration longer than 5 days (1st trimester timed collection vs. 1st trimester fetal demise). Consistent with interrupted infection at this early timepoint, there was no discernable histopathology or inflammation in the fetus (data not shown). Tissues at the maternal-fetal interface demonstrated necrosuppurative arteritis in the decidua and multifocal suppurative necrosis of placental septa and anchoring villi, with a laterally dissecting hemorrhage and focal abscessation of a basal vein. Of note, cy23.1 tested positive for Lm in both fecal and blood cultures the day after inoculation. In contrast, cy27.2 had no maternal fecal shedding or bacteremia, and tissue samples at day 10 post-inoculation yielded no positive cultures and no evident histopathology (Figure 1A; Table 2).

**Table 2:** Histopathology score chart.

**Treatment (Tissue +/–)**	**Trimester**	**ID**	**Maternal-Fetal Interface**						**Fetal**					**TOTAL**
			**Lymphocytic and neutrophilic deciduitis**	**Decidual fibrinoid necrosis**	**Placental villous necrosis**	**Neutrophilic villitis**	**Neutrophilic chorio-amnionitis**	**MFI score**	**Funisitis**	**Hepatitis**	**Splenitis**	**Pulmonary congestion with neutrophils**	**Fetal score**	
Control	1	cy24c						0					0	0
Control	1	cy27c						0					0	0
Control	1	cy26c	x					1					0	1
Control	1	cy35c	x		x			2					0	2
Lm (+)	1	cy23.1	x		x	xx		4					0	4
Lm (+)	1	cy30.2	xxx			xxx	xx	8					0	8
Lm (+)	1	cy21.1	xx	x	x	xx	x	7		xxx		x	4	11
Lm (+)	1	cy25.1	xx	xx	x	xx	xxx	10	x	xxx			4	14
Lm (+)	1	cy19.1	xx	xx	xxx	xxx	x	11	x	xx		x	4	15
Lm (–)	1	cy27.2						0					0	0
Lm (–)	1	cy26.2	x	x	n/a	n/a	n/a	n/a	n/a	n/a	n/a	n/a	n/a	n/a
Lm (–)	2	cy23.2	x^*^			x		2					0	2
Lm (–)	3	cy26.1	x^*^			x	x	3					0	3
Lm (–)	3	cy29.1	xx^*^		xx			4			x		1	5
Lm (–)	3	cy27.1	xx	xx	xx			6		x			1	7
Lm (–)	3	cy29.2	x					1					0	1
Lm (–)	3	cy25.2	x^*^					1					0	1
Lm (+)	3	cy39.1				x		1					0	1
Lm (–)	3	cy20.1			x			1		x			1	2
Lm (–)	3	cy30.1			xx	xx		4				x	1	5
Lm (+)	3	cy34.1	xx	xx		xx		6		x			1	7
Lm (+)	3	cy31.1	xxx			xx	xxx	8		x		xxx	4	12
Control	3	cy35c						0					0	0
Control	3	cy33c		x				1					0	1
Control	3	cy38c		x				1					0	1
Control	3	cy31c		x				1					0	1

*Histological scores for samples from the maternal-fetal interface and fetal tissues are presented as the sum of discrete pathologies noted by WNPRC board-certified veterinary pathologists on examination of H&E stained sections. Cases are organized by cohort and trimester at the time of tissue collection. ID ending in “c” indicates control, “1” indicates first pregnancy, and “2” indicates second pregnancy. Case cy26.2 had no identifiable uterine contents following spontaneous abortion, and n/a indicates these samples could not be analyzed for discrete histopathology*.

* *Presence of plasma cells (indicative of a chronic inflammatory response)*.

Score criteria are as follows:

*X, mild (rare single scattered cells throughout the histologic section or multiple clusters of 4–10 cells; findings impact 0–25% of thesection)*.

*XX, moderate (1–2 clusters of 10–20 cells or diffuse cell infiltration; findings impact 26–50% of thesection)*.

*XXX, severe (1–2 clusters of more than 20 cells; findings impact more than 50% of thesection)*.

### Third Trimester Infection

We assessed third trimester pregnancy outcomes following infection with the same clinical strain of Lm. Two of three monkeys given 10^7^ CFUs Lm in the third trimester of pregnancy had a stillbirth between 10 and 14 days post-inoculation. A fourth monkey received 10^8^ CFUs and gave birth prematurely at 4 days post-inoculation. All three of these cases had Lm+ maternal fecal shedding, bacteremia, and tissue colonization (Figures 1C, 5). The most severe pathologic changes were seen in the stillbirth cases, characterized by neutrophilic deciduitis, suppurative villitis, and chorioamnionitis at the maternal-fetal interface, and fetal hepatitis and pulmonary congestion with neutrophils (Figure 2C). The preterm infant had Lm-positive cultures only within the amniotic membranes, placenta, and decidua, and no discernible inflammatory reaction or pathologic changes in the fetus (Table 2). The lack of fetal colonization is likely due to the occurrence of preterm birth before Lm could traffic to the fetal compartment, consistent with our observation that cy23.1 had a comparatively low fetal bacterial burden at post-inoculation day 5 (Figure 5). One dam (cy20) showed no evidence of maternal infection and continued to term uneventfully, but examination of tissues after surgical collection demonstrated villous necrosis of the placenta as well as fetal hepatitis, neither of which have been noted in age-matched control tissues. All third trimester placentas had variable mineralization, also seen in healthy controls, and the degree of mineralization most strongly associated with increasing gestational age. Focal placental infarction with few scattered neutrophils and focal areas of decidual necrosis is also an incidental finding in the third trimester (Figure 2D). Areas of infarction and mineralization are a normal consequence of cell death and ischemia due to the nature of the placenta as a temporary fetal organ that undergoes senescence as it nears parturition.

## Discussion

It is known that listeriosis causes adverse pregnancy outcomes, including miscarriage, stillbirth, and neonatal infection (Hof, 2003; Lamont et al., 2011; World Health Organization, 2018). Our research aims to clarify the course of infection, as well as the histological and cellular sequelae, which cannot be readily studied in human pregnancy. We have previously described the course of infection following a first-time exposure in the first trimester of pregnancy in a non-human primate model, resulting in acute infection and loss of the pregnancy between 1 and 2 weeks post-inoculation (Wolfe et al., 2017). Here we employed the same clinical isolate of Lm to determine how a lower initial dose impacts disease pathogenesis and pregnancy outcomes, if the virulence of our strain of Lm depends on gestational age, and whether or not prior exposure influences the immune response with reinfection in a subsequent pregnancy. Our previous study showed that the first trimester of pregnancy is keenly sensitive to infection, which caused fetal demise in 4 out of 4 monkeys following inoculation with 10^7^ CFUs Lm. With the lower dose given in this study, we hypothesized that we would observe a long incubation period followed by acute fetal infection, which is thought to occur in human outbreaks based on retrospective surveys, clinical cases and estimated exposure dates (Goulet et al., 2013). However, we found that inoculation with 10^6^ CFUs Lm in the first trimester did not result in latent maternal infection, nor in acute or chronic fetal infection. All 3 monkeys progressed to term C-section without adverse events. Infant weights remained comparable to controls, and there were no gross abnormalities. None of the sampled tissues had Gram-positive stained or culturable Lm. Interestingly, however, histological examination of tissues following surgical collection revealed lesions consistent with chronic inflammation and intrauterine bleeding, not found in gestational age-matched control samples, as well as a shift in the placenta toward a pro-inflammatory cytokine profile.

We next examined the impact of gestational age on infection susceptibility and pregnancy outcomes. Following the same experimental paradigm as before, we observed an adverse outcome in 3 out of 4 monkeys given 10^7^-10^8^ CFUs Lm in the third trimester. Two spontaneously aborted at 10- and 14-days post-inoculation with significant inflammation of the maternal and fetal tissues, a tissue bacterial burden comparable to that seen in the first trimester, and histological evidence of fetal hepatitis and pneumonia. The third (cy39.1) delivered a premature infant 4 days post-inoculation. Although the fourth (cy20.1) continued to term C-section, there was evidence of fetal distress *in utero* and abnormal placental necrosis. At 10^7^ CFUs Lm, our combined studies show a survival rate of 1/3 in the third trimester and 1/5 in the first trimester. Our findings of fewer stillbirths yet still severe outcomes in later pregnancy are consistent with reports that fetal survival odds increase with gestational age, potentially due to an improved fetal immune response (Wadhwa and Smith, 2017). It will be intriguing for future studies to assess the development and adult health of infants known to have survived a pregnancy impacted by listeriosis.

Despite moderate to severe inflammation at the maternal-fetal interface, there was frequently minimal to absent fetal immune response detected by histologic examination (Table 2: cy23.1, cy30.1, cy34.1, cy26.1, cy26.1, cy27.1). The capacity for a fetal immune response emerges as pregnancy progresses with the development of hematopoietic and lymphoid organs (Chougnet, 2018). Differentiated immune cells, including neutrophils, macrophages, B cells, and T cells, can be observed by the late second to early third trimester, but these cells appear to be geared toward immunosuppression rather than defense against non-self-entities, and evidence suggests that the fetal immune system functions distinctly differently from that of adults as well as newborns, given its *in utero* niche and need to coexist with the maternal immune system (Makori et al., 2003; McGovern et al., 2017). An absence of fetal inflammatory response in the first trimester can be attributed to early gestational age and a lack of infiltration by maternal immune cells. At later stages of pregnancy, this may be due to fetal death or expulsion of uterine contents occurring before tissue-level changes caused by a cellular defense response can be observed. Similar to our early pregnancy study, we observed a fairly short incubation period of 4–14 days, which may be strain-dependent.

To determine if prior exposure to Lm confers protection or causes sensitization to subsequent exposures, we re-inoculated dams with an equivalent or higher dose of the same clinical isolate of Lm at approximately the same gestational day during a second pregnancy. We hypothesized first-time exposure would elicit a maternal immune response that would provide subsequent protection. Re-exposure resulted in a lower rate of pregnancy loss than first-time exposure, which is consistent with observations in livestock (Fensterbank, 1987), however we did not identify a clear association between initial response and secondary response that would have allowed us to predict the pregnancy outcomes of specific individuals. For example, cy25 lost her first pregnancy to Lm infection and then carried her second pregnancy to term despite reinfection, suggesting protection. In contrast, cy23 also had fulminant tissue colonization and maternal bacteremia in her first pregnancy, yet lost her second pregnancy to Lm infection. Cy30 and cy26 each had successful first pregnancies but miscarried their second, while cy29 successfully carried both pregnancies to term despite culture-positive maternal Lm infection each time. In total, re-exposure resulted in fetal demise in 3 out of 5 cases. Two of these cases (cy30.2 and cy23.2) could be diagnostically attributed to listeriosis. We did not recover Lm from the third case (cy26.2). While this case cannot be diagnostically attributed to listeriosis because all bacterial cultures and histologic stains for Lm were negative, it is possible that Lm does not need to directly infect the reproductive tract to induce adverse pregnancy outcomes. Peripheral immune cell activation following maternal infection may induce a signaling cascade of pro-inflammatory cytokines or other cellular responses contributing to the incidence of spontaneous abortion and preterm birth (Rowe et al., 2012).

The rate of miscarriage in this study exceeds the rate of spontaneous abortion at WNPRC's breeding colony of macaques, which were not used in these experimental studies. The breeding colony at WNPRC serves as an excellent control for infection studies because these animals are housed indoors and protected from environmental exposures. According to colony management records, the rate of spontaneous abortion in the colony per year is around 1 in 25 pregnancies. Less than 1% of these could be attributed to spontaneous ascending bacterial infection associated with fecal or vaginal flora. Prior to initiation of this study, there were no positive Lm culture results in the WNPRC database, which spans 35+ years of clinical and pathological results. All WNPRC animals and aborted tissues receive gross and histologic evaluation with appropriate bacteriological and viral testing. Additionally, our studies on listeriosis to-date have included a total of 10 designated control monkeys who underwent the same treatments and procedures as our experimental animals, except that they did not receive Lm in the inoculum. None of the control pregnancies in our current or previous studies have resulted in an adverse outcome, which suggests that maternal exposure to Lm, even without detectable reproductive tract colonization, has a detrimental impact on pregnancy health.

A preliminary assessment of cytokine expression in placenta samples from our cohorts shows that exposure to Lm alters the balance of pro- vs anti-inflammatory mediators at the maternal-fetal interface. Principle component analysis (PCA) can be used to visualize the degree of variability between sample sets and is particularly useful in demonstrating general relationships among subgroups based on multivariate data (Helmy et al., 2012). Because cytokines are not expressed in isolation, rather many are pleiotropic, act upon one another, and have the capacity to be pro-inflammatory or anti-inflammatory depending on the context and presence other cytokines, we therefore wanted to perform an exploratory cluster analysis to evaluate how much variation between our cohorts could be identified based on their overall pattern of cytokine expression. On PCA plot, Lm samples cluster apart from control samples. Given the inflammatory tissue response induced by Lm infection, this confirms expectations. Notably, Lm pregnancy loss samples had reduced IL-1RA expression, which has been linked to an impaired primary immune response and increased susceptibility to Lm infection (Irikura et al., 1999). Placenta samples from dams who received Lm yet went on to have uneventful pregnancies (Lm survival) clustered more closely to Lm pregnancy loss samples than to controls. Intriguingly, Lm survival samples had greater IL-1RA expression than Lm pregnancy loss samples, yet lower than controls. This suggests that the histopathology observed in the Lm survival cohort may be underpinned by responsive cell signaling. On the same plot, placentas from dams re-exposed to Lm in a subsequent pregnancy clustered apart from all other groups, suggesting a distinct secondary response. Hierarchical clustering based on cytokine expression demonstrates that exposure to Lm alters the chemical milieu of the placenta even in cases without adverse pregnancy outcomes. It will be enlightening to confirm these trends pending a larger study with additional samples, and to further evaluate this dataset in relation to cytokine signaling from the maternal decidua and fetal amniotic membranes. We cannot say if the Lm survival cohort's sterile yet inflammatory intrauterine environment would have an impact on infant development, behavior, or health in later life, as this study was not designed to explore that question. Retrospective studies on the long-term morbidity and neurodevelopment of survivors of prenatal listeriosis are mixed (Evans et al., 1984; Bortolussi and Mailman, 2011) and more definitive studies assessing prenatal programming from a pregnancy impacted by maternal Lm infection would be valuable.

Diagnosing Lm infection prior to an adverse event such as stillbirth or preterm birth continues to be difficult and inconsistent based on patient presentation and laboratory results (Charlier et al., 2014). A retrospective study at a hospital in Israel found that listeriosis is simultaneously underdiagnosed and overtreated (Fouks et al., 2018): over the course of five years, more than one hundred pregnant women who received a diagnosis of suspected listeriosis did not subsequently have a Lm+ culture, while 7 patients who had culture-confirmed Lm had not initially received a diagnosis of suspected listeriosis. One of the advantages of our model is that we can assess infection outcomes given a known bacterial quantity and timing of exposure. Although we directly administered a known amount of Lm to the maternal gastrointestinal tract, we note inconsistent fecal shedding of bacteria, clinical signs, and disease course. In some cases, we were unable to grow colonies on blood agar plates even though microscopy showed Gram-positive rods in tissues, and in other cases we did not observe Gram-positive rods despite Lm-positive cultures. In several cases, we had Lm-positive maternal samples, yet did not recover Lm from fetal or maternal-fetal interface samples through assay, culture, or cold enrichment. It has been reported that Lm is capable of entering a viable but non-culturable (VBNC) state (Highmore et al., 2018) which could complicate diagnostics, and Gram-staining can yield false results if artifacts are introduced or errors are made in specimen collection and processing (Samuel et al., 2016). Another cause for discrepancy with Lm detection across samples may be the characteristically multifocal pattern of listerial colonization: within the same tissue, we observe infiltration of some regions but not others. As we collect biopsies, it is possible that one contains a focus of Lm while another does not. In cases with maternal bacteremia and extensive tissue colonization, we had no difficulty identifying Lm; rather it was the cases in which there was light maternal fecal shedding and minimal colonization of maternal organs that proved more ambiguous. In these cases, we hypothesize that Lm was restricted from the intrauterine compartment, and that histologically-evident placental and fetal pathologies resulted from an immune response to infection, as opposed to direct infection. Maternal bacteremia was our strongest predictor of an adverse outcome and associated fetal histopathology. The pregnancies of dams who became bacteremic consistently ended in acute fetal demise shortly after initial detection of bacteremia, and all of these cases had bacterial colonization of the maternal-fetal interface, in contrast to dams with positive fecal cultures but no bacteremia, indicating that hematologic trafficking is critical for Lm to reach the reproductive tract. Identifying accurate and reliable markers of early infection that can be tested *in utero* remains an important goal to prevent the unnecessary use of antibiotics while at the same time ensuring that patients genuinely at risk of pre- and perinatal listeriosis can be promptly recognized and treated.

Based on our findings, prior maternal exposure does not appear to be predictive of disease susceptibility or pregnancy outcome, nor does the timing or duration of maternal shedding of Lm. Interindividual variation in response to transmissible disease is notable in humans, and no doubt factors into the range of outcomes observed in nonhuman primates as well (Casadevall and Pirofski, 2018). Confounding factors such as environment, diet, exercise, comorbidities and prior medical treatment plausibly may be ruled out given the controlled conditions of indoor housing and colony management at WNPRC facilities. We documented fetal sex and maternal age, weight, and body temperature, and these do not appear to correlate with pregnancy outcome. Maternal temperatures were, on average, mildly elevated following Lm inoculation, and two dams had clinical fever, but this was neither pathognomonic nor predictive of infection. We took into account the range of maternal body size by calculating the Lm dose per kilogram, and individual dams proved susceptible to different doses. Some, like cy34.1, had pregnancy loss with 2^6^ Lm/kg, whereas cy30.1 tolerated 4^6^ Lm/kg without pregnancy loss. Like humans, monkeys are genetically diverse and it is possible that disease resilience is driven by genomic differences or microbiome composition (Namasivayam et al., 2019), or that social dynamics play a role in immune programming (Snyder-Mackler et al., 2016). Individual determinants of disease remain an important area of study.

Although we observed fewer adverse pregnancy outcomes in our cohort of monkeys that received a lower dose of Lm, we cannot conclude and do not suggest that we have found any safe level of exposure to Lm. It is evident that different bacterial strains, environmental conditions, and individual susceptibility can alter Lm virulence and infection kinetics (Vázquez-Boland et al., 2001; Smith et al., 2008). Our study instead offers evidence that maternal Lm infection which spares the fetus still impacts the placenta, creating a more pro-inflammatory intrauterine environment, and reiterates that Lm is a pathogen of serious consequence. Ongoing studies from these same cohorts examining the maternal and fetal cellular response to listeriosis, including transcriptomic changes, post-transcriptional modifications, and host microbiome shifts, aim to shed light on the determinants of disease susceptibility vs. resilience.

## Data Availability

WNPRC has a policy of sharing data and materials when scientifically relevant. The datasets for the current study are available upon reasonable request. Requests to access the datasets should be directed to TG (golos@primate.wisc.edu) and to HS (hsimmons@primate.wisc.edu).

## Ethics Statement

The animal study was reviewed and approved by University of Wisconsin Institutional Animal Care and Use Committee.

## Author Contributions

BW, CC, and TG contributed to the conception and design of the study. BW organized experiments, quantified bacterial burden, produced figures, and wrote the first draft of the manuscript. HS and AM collected, read, and scored histological specimens. HS photographed H&E sections. AK curated, scanned, and analyzed Gram-stained specimens. BW, HS, and TG wrote sections of the manuscript. All authors contributed to manuscript revision, read, and approved the submitted version.

### Conflict of Interest Statement

The authors declare that the research was conducted in the absence of any commercial or financial relationships that could be construed as a potential conflict of interest.

## Abbreviations

CFUs: colony forming units
GD: gestational day
Lm: *Listeria monocytogenes*
MFI: maternal-fetal interface
PCA: principle component analysis
WNPRC: Wisconsin National Primate Research Center.
